# Pooling for SARS-CoV2 Surveillance: Validation and Strategy for Implementation in K-12 Schools

**DOI:** 10.3389/fpubh.2021.789402

**Published:** 2021-12-17

**Authors:** Alexandra M. Simas, Jimmy W. Crott, Chris Sedore, Augusta Rohrbach, Anthony P. Monaco, Stacey B. Gabriel, Niall Lennon, Brendan Blumenstiel, Caroline A. Genco

**Affiliations:** ^1^Department of Immunology, Tufts University School of Medicine, Boston, MA, United States; ^2^Office of the Vice Provost of Research, Tufts University, Boston, MA, United States; ^3^Jean Mayer United States Department of Agriculture (USDA) Human Nutrition Research on Aging at Tufts University, Boston, MA, United States; ^4^Tufts Technology Services, Somerville, MA, United States; ^5^Tufts University, Medford, MA, United States; ^6^Broad Institute of MIT and Harvard, Cambridge, MA, United States; ^7^Graduate Program in Immunology, School of Graduate Biomedical Sciences, Tufts University School of Medicine, Boston, MA, United States; ^8^Molecular Microbiology, School of Graduate Biomedical Sciences, Tufts University School of Medicine, Boston, MA, United States

**Keywords:** pooled testing methodology, COVID-19, SARS-CoV-2, RT-PCR assay, screening

## Abstract

Repeated testing of a population is critical for limiting the spread of the SARS-CoV-2 virus and for the safe reopening of educational institutions such as kindergarten—grade 12 (K-12) schools and colleges. Many screening efforts utilize the CDC RT-PCR based assay which targets two regions of the novel Coronavirus nucleocapsid gene. The standard approach of testing each person individually, however, poses a financial burden to these institutions and is therefore a barrier to using testing for re-opening. Pooling samples from multiple individuals into a single test is an attractive alternate approach that promises significant cost savings—however the specificity and sensitivity of such approaches needs to be assessed prior to deployment. To this end, we conducted a pilot study to evaluate the feasibility of analyzing samples in pools of eight by the established RT-PCR assay. Participants (1,576) were recruited from amongst the Tufts University community undergoing regular screening. Each volunteer provided two swabs, one analyzed separately and the other in a pool of eight. Because the positivity rate was very low, we spiked approximately half of the pools with laboratory-generated swabs produced from known positive cases outside the Tufts testing program. The results of pooled tests had 100% correspondence with those of their respective individual tests. We conclude that pooling eight samples does not negatively impact the specificity or sensitivity of the RT-PCR assay and suggest that this approach can be utilized by institutions seeking to reduce surveillance costs.

## Introduction

Shutdowns resulting from an effort to control the ongoing COVID-19 pandemic have had a detrimental effect on the education of K-12 school children, especially those in at-risk socioeconomic groups and those with special needs (1, 2). For example, clinicians note significant weight gains and precipitation of anxiety disorders during COVID-19 (3). Educational attainment is an important predictor of future health, mental state, and socio-economic outcomes (4). Returning children to school in a safe and affordable way is a high priority. In addition to appropriate safety precautions like social distancing and mask wearing, testing of all individuals in a population at regular intervals can reduce transmission via early detection of asymptomatic or pre-symptomatic infectious viral carriers who can be a source of transmission.

Along with limited laboratory equipment, reagents, and resources, the cost of repeated individual testing presents a substantial challenge. One potentially more efficient testing strategy is sample pooling, a broadly developed mathematics subfield wherein samples from individuals are grouped together and tested as a single unit with a single output. Pooled testing can reduce the number of tests performed by a factor of the pool size, reducing cost as well as laboratory throughput demands. The simplest form of pooling is known as Dorfman or two-stage hierarchical pooling (5). By this method, each pool contains a set number of samples. Each sample is tested once as part of a pool, and again as an individual test only if the pool tests positive. Other, more complex methods assign a single sample to more than one pool to better predict positive samples, thus reducing the number of individual retests necessary (6–8). Dorfman pooling for SARS-CoV-2 RT-PCR testing has been shown to be effective at the level of eight samples per group (9).

The CDC-approved, commonly used diagnostic test for SARS-CoV-2 infection employs reverse transcription-polymerase chain reaction (RT-PCR) to detect viral RNA present in mucosal samples immersed in a liquid buffer (10). Sample pooling has been proposed and validated by a number of groups, with the pooling step performed at various points in the diagnostic workflow e.g. (1) extracting RNA from multiple swabs in a single container, (2) combining extracted RNA into a single container for cDNA synthesis, or (3) pooling the cDNA after production on an individual basis. Benefits of pooling later in the process include equal amounts of input per subject and rapid retesting of positive pools if individual samples are stored. However, the earlier pooling occurs in the process the greater cost-savings in reagents. Additionally, direct pooling of individual swabs into a single tube can be done at the site of testing, effectively saving time, labor, and materials. Because of the financial benefits, the Food and Drug Administration (FDA) has already issued the first Emergency Use Authorization (EUA) for pooled testing of SARS-CoV-2 for pools of up to 4 samples (11).

Importantly, for SARS-CoV-2 pooled testing there are several variables including sampling site, swab storage (wet or dry), pool size and timing of pooling (before or after RNA extraction) each of which may affect the reliability of the assay. Although others have established pooled testing using smaller pools or wet storage [e.g., (9, 12–16)], it is critical to validate each specific method in its entirety, especially when dealing with life-threatening illnesses. Recently, the Broad Institute has established a clinical laboratory to perform RT-PCR from RNA extracted from anterior nares dry swabs. Here, we present a proof-of-concept pilot study intended to validate the efficacy of pooled SARS-CoV-2 testing of up to 8 dry swabs when compared with individual testing.

## Methods

This study was reviewed and approved by the Institutional Review Board of Tufts University (STUDY00000979: Pooled Testing). Written informed consent was obtained from all participants. Eligibility criteria included being a member of the Tufts University faculty, staff, or student populations as outlined by risk of exposure to COVID-19 (https://coronavirus.tufts.edu/testing-at-tufts), ages 18–100.

Tufts University has implemented a comprehensive COVID-19 testing program to enable the safe return of students to campus. Students, faculty, staff, and researchers are required to test 1–2 times per week depending on their residential arrangement, frequency and nature of campus use, interactions with each other, and exposure to patients or the general public.

For this program, individuals reported to various testing locations and self-swab their anterior nares with a single sterile soft-tip swab (Puritan Medical Products LLC) which is then deposited into a prelabelled plain vacutainer (Beckton Dickinson). Samples were couriered to the Broad Institute 4–6 times per day and analyzed by the established CDC RT-PCR assay with fluorescent detection (FAM-labeled probes) (11). This test uses primer/probe sets developed by the CDC that target two viral gene targets in the Nucleocapsid gene of SARS-CoV-2, N1 and N2, and an internal control gene, RNase P (RP).

In September and October of 2020, we obtained 2,032 samples from members of the Tufts University community undergoing routine screening. Individuals were not restricted from participating in the study multiple times. Upon arrival at the testing site, participants were provided with two swabs and instructed to swab one per nostril and deposit each in a separate vacutainer. The method of collection was the dry swab method described elsewhere (11). At the end of each study day, one sample per individual was sent for regular individual testing while the other was processed for pooled testing. For pooling, individual samples were removed from their vacutainer and placed in a 50 mL Falcon tube (Thermo Fischer Scientific) and shipped to the lab dry. Upon receipt at the laboratory, tubes were de-capped and guanidinium thiocyanate lysis buffer added −5 ml for pooled tubes and 1 ml for individual tubes. After shaking tubes on orbital shaker for 15 min, 50 μl of swab buffer (for both pooled and individual tests) underwent bead-based RNA extraction using the MagMax Viral extraction kit (Thermo Fisher Scientific). qPCR was performed using TaqPath qPCR mix (Thermo Fisher) on Quant Studio 7 (Thermo Fisher) instrument. A test is deemed “invalid” if there is no signal (>40 Ct) for either the RP human assay or the N1/N2 targets. A test is called “negative” when the RP Human target is detected (<40 Ct) and no Covid targets are detected (>40 Ct).

The limit of detection for the RT-PCR assay is 60 copies per reaction (17) which equates to 50 μl of a 1,200 copy/ml swab solution. Thus, to generate a 1,200 copy/ml solution in 1 ml (individual testing) the swab would need to provide 1,200 viral copies. To generate the same concentration (1,200/ml) in a 5 ml solution (pooled testing) the positive swab would need to have 6,000 copies. The CDC has determined that mutations present in available SARS Cov-2 sequences as of 6 Jun 2021 (including the B.1.1.519 variant) are not predicted to reduce the sensitivity or specificity of the primer/probe set (10).

One hundred and fifty-eight pools were processed as groups of eight samples. Eleven of these pools were made up of seven community samples plus an additional sample obtained from individuals under investigation based on a previous positive SARS-CoV-2 RT-PCR test. Of these 11 samples, only two were positive in this study. The remaining nine were likely cases of prior infection. Another 114 pools were made up of seven community samples plus an eighth laboratory-generated positive sample prepared by the Broad Institute as described in their EUA (11). Briefly, these samples were generated by re-suspending nasal swab material from known positive cases and pipetting it onto new swabs as previously described (18). Approximately 102,164 copies of the virus were pipetted on to each swab and allowed to air dry. One laboratory-generated positive swab was added to each Falcon tube containing the seven community samples and the pools were processed in 5 ml of buffer as described above. Because 50 μl of the buffer is used for RNA extraction it is estimated that approximately 1,022 viral copies went into the extraction. When considering the N2 amplicon, our spiked samples returned a Ct of 29.4 ± 0.7 (*N* = 105). For real-world positives, the Ct values range from 9.0 to 39.5, although approximately two-thirds have a Ct below 28. We therefore conclude that the amount of virus spiked onto our lab generated positives is physiologically relevant and indeed lower than the majority of real-world samples.

All samples were analyzed within 24 h of collection.

## Results

**Figure 1:**
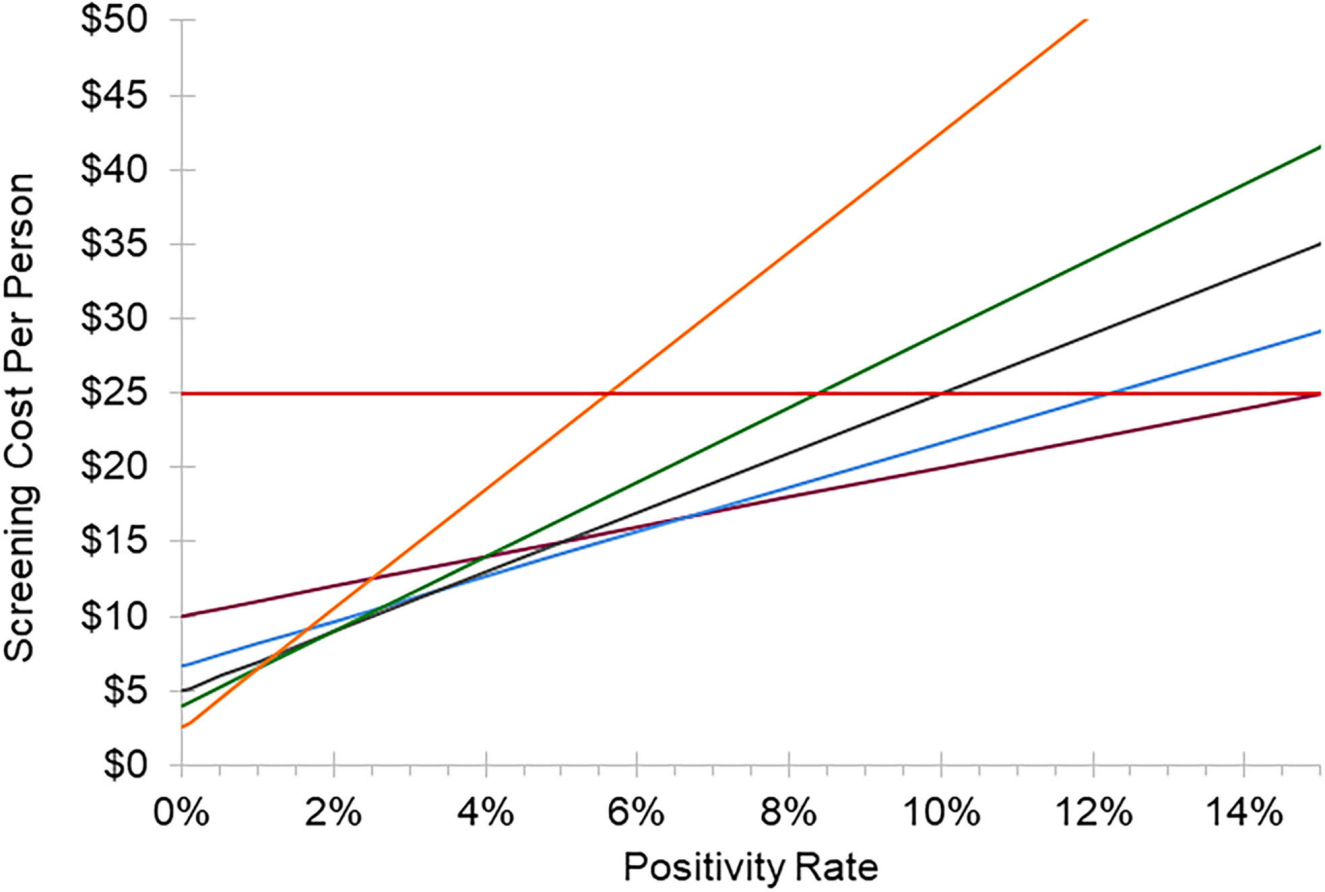
Cost of individual vs. pooled testing. Cost per person of testing as a function of the percent positivity of the population tested. This model assumes an individual test cost of $25 and a pooled test cost of $40 due to additional processing steps required. Red line: individual testing, maroon line: pool size of 4, blue line: pool size of 6, gray line: pool size of 8, green line: pool size of 10, orange line: pool size of 16. Equation for pooled testing: ($40/pool size) + (% positivity × pool size × $25).

For this study, we selected eight samples per pool for financial and logistical reasons. Financially, the cost and efficiency of various pooling ratios changes as a function of the percent positivity in the population (Figure 1). A pool size of eight was the most cost effective per person for positivity rates ranging from 2.22 to 2.86%. At positivity rates lower than 2.22% it is still cost-effective when compared with pools of 9–10 (at a positivity rate of 0.00%, a pool of 8 costs $5.00/person while a pool of 10 costs $4.00/person). Large pool sizes are more efficient and cost effective at lower community positivity rates, but the larger the pool, the more rapidly it increased the cost as the percent positivity increased (Figure 1). It is critical that the pool size is flexible to accommodate varying positivity rates in the population. To ease scalability and adoption, it was also important in this study to (1) minimize the operational transition from individual to pooled testing, and (2) pool samples at the site of collection using standardly available materials. Because of these factors, including limitations of rehydration volumes and tube sizes, we did not consider pools larger than 10.

**Figure 2:**
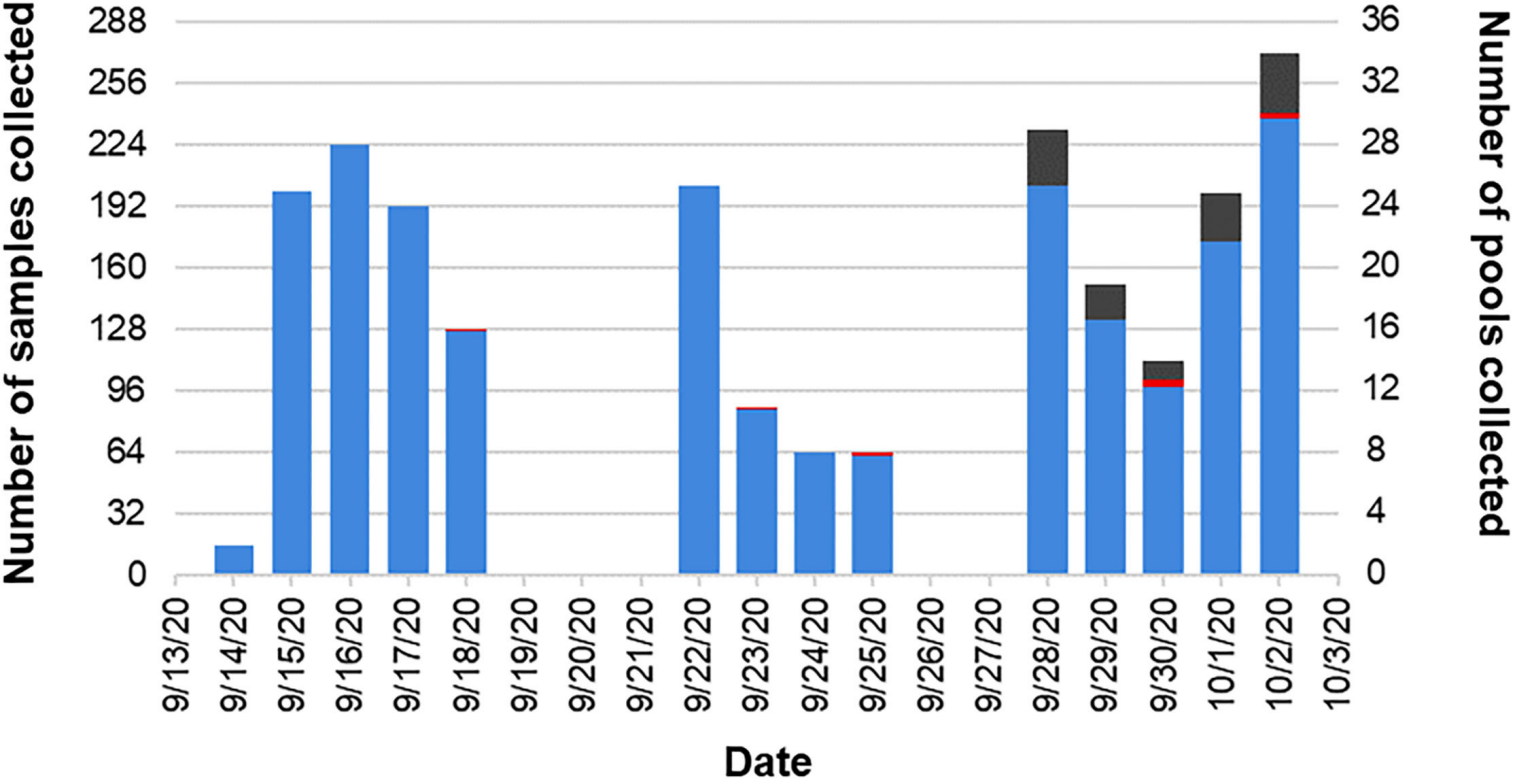
Pooled pilot study timeline. Two thousand and thirty-two pairs of samples were collected over a 3-week period. Two thousand and twenty-one of those pairs were collected from the Tufts University community (blue). The other 11 pairs were collected from individuals under investigation and in quarantine due to possible exposure to SARS-CoV-2 (red). During the third week of the study, the low positivity rate in the Tufts community necessitated the inclusion of laboratory-generated positive samples (gray).

**Figure 3:**
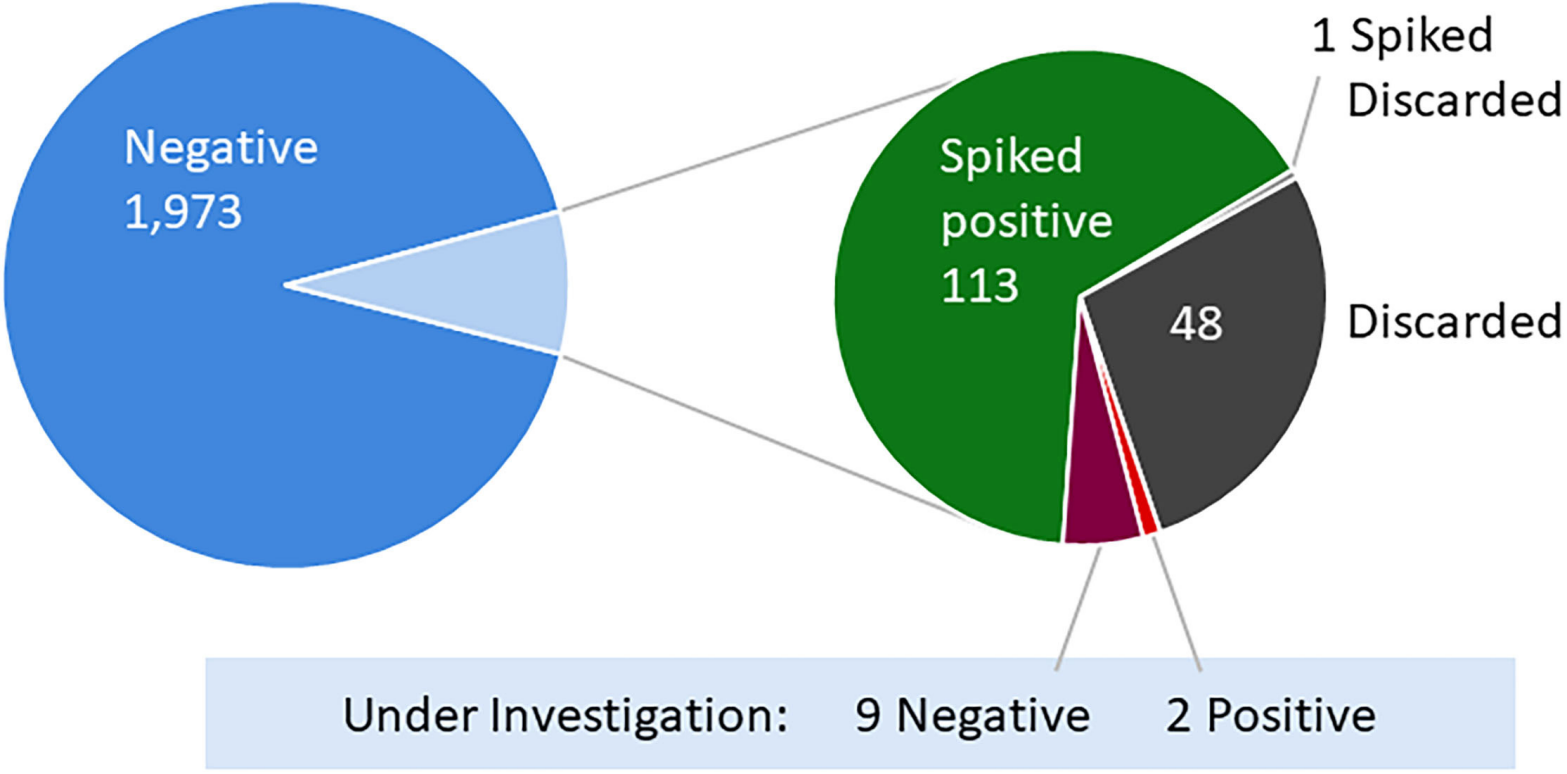
Individual sample results of SARS-CoV-2 RT-PCR testing. Of the 2,032 community-sourced and 114 laboratory-generated pairs of samples, one sample from each pair was tested as an individual using the established SARS-CoV-2 RT-PCR testing protocol. Eleven of the sample pairs were collected from individuals under investigation and in quarantine due to possible exposure to SARS-CoV-2 (red and maroon).

Over 3 weeks we collected 2,032 pairs of samples from 1,576 students, faculty, and staff already subject to regular COVID-19 surveillance testing at Tufts University (Figure 2). Of these, 1,973 samples came back negative (Figure 3). Of the eleven samples taken from individuals under investigation due to possible SARS-CoV-2 exposure, only a single individual was responsible for the two positive samples from our community-based sampling. The study was originally designed to assess sensitivity by collecting only natural positive cases. However, the very low positivity rate within the Tufts community made it impossible to obtain our target number of pools with at least one positive within a reasonable period of time. Thus, to obtain sufficient positive pools we implemented the artificial spiking protocol described in the methods, wherein pools of seven swabs obtained from community members were supplemented with an additional swab generated from known positive samples prior to RNA extraction. In testing individual samples, we observed a collection failure rate of 2.36% (48 samples) (Figure 3). Of these, 24 samples were discarded due to a single laboratory cataloging error, four were lost on site, eleven were discarded due to improper collection methods (excessive material on the swab, swab inverted, or contamination with blood), and nine produced an invalid RT-PCR result, likely due to a lack of RNA present. For unknown reasons, one of the laboratory-generated spiked samples also produced an invalid RT-PCR result.

To evaluate the efficacy of pooling samples, results of the individual samples were compared with results of their respective pools (Table 1). While 272 sample pools were prepared and successfully analyzed, data from 32 pools was discarded due to a failed individual assay for one or more of the paired samples in the pool as described above. Thus, data for a total of 240 pools were included−133 with samples of unknown SARS-CoV-2 status and 107 spiked with a known positive. We observed 100% congruency between the approaches; all pools containing a swab whose individual counterpart had tested positive also tested positive and all pools for which individual samples were all negative also tested negative.

**Table 1:** Comparison of pooled with individual sample results.

	**1 positive sample in pool**	**All samples negative in pool**
Pooled positive result	107	0
Pooled negative result	0	133

*Two hundred and seventy two pools were tested. Thirty two pools were discarded because one or more of the corresponding individual sample tests was invalid, leaving 240 pools which corresponding exactly with the individual results. 107 positives includes 105 lab generated pus 2 natural positives*.

For both pooled and individual assays, samples were deemed negative when the Ct value for the N1 and N2 amplicons were >40 and the human RP gene was <40. For the 107 positive pools (105 spiked +2 natural) the Ct values for the N1 and N2 amplicons ranged from 27.6 to 30.5 (Mean = 29.0 ± 0.06) and 28.1 to 32.2 (Mean = 29.4 ± 0.07), respectively. When assayed individually, these positive samples returned Ct values of 26.5–28.2 (Mean = 27.5 ± 0.04) and 26.9–29.5 (Mean = 28.0 ± 0.1) for the N1 and N2 amplicons, respectively. The average Ct values of positive samples were on average 1.4 and 1.5 cycles higher for the pooled samples compared to the matching individual sample, for N1 and N2, respectively—which is to be expected because the pooled samples are rehydrated in larger volume of buffer than individual samples (5 vs. 1 ml) while the same volume (50 μl) of solution is used for the assay in both cases and mimics observations from other studies (19).

## Discussion

Pooled sample testing is an effective strategy to reduce the cost of regular screening testing during pandemics. We conducted a pilot study to evaluate the validity of pooling anterior nasal swabs for the detection of SARS-CoV-2 by RT-PCR. While others have validated pooling of “wet swabs” (14–16) this is the first evaluation of pooled testing using eight dry swabs. Dry swabbing presents a substantial benefit over wet by saving reagents, thus saving money as well as reducing vulnerability to the supply-chain issues that have interfered with testing capacity during the COVID-19 pandemic.

Potential concerns with pooled testing include the dilution of sample resulting in false negatives and the accumulation of contaminants resulting in false positives. We observed a 100% congruence between the results of the pooled and individual analyses—indicating that there is no loss of specificity or sensitivity when performing SARS-CoV-2 screening from pools of eight dry swabs compared to individual analyses. Given that the clinical specificity and sensitivity of the CDC's 2019-nCoV Real-Time RT-PCR Diagnostic Panel are 100% (13/13; 95% CI: 77.2–100%) and 100% (104/104; 95% CI: 96.4–100%) (10), respectively, this provides high confidence in identifying all positive individuals using pooling.

One clear limitation of the current study was the reliance of laboratory-generated positives due to the low positivity rate in our population. Although our positive spike was generated from a high titer clinical sample, our study could have further benefitted from using spikes with varying viral loads as opposed to a single concentration.

A recent publication by members of the Broad Institute established a reduction in sensitivity that is roughly linear with the log of the dilution factor employed by simulating pooling under varying population prevalence, pool size, and population size (18). Specific recommendations for pooled testing from the FDA have only recently become available (20), and further investigation is necessary to evaluate its efficacy for specific circumstances. For this study, we selected eight samples per pool for logistical and financial reasons described above. Our protocol has a limit of 10 samples because it calls for rehydrating swabs as a pool. This minimizes labor costs since all subsequent steps can follow the established semi-automatic individual testing workflow. Crone et al. (21) also validated pools of 8 and 10 and estimated savings of 28–90% on each of the reagents and consumables used in the assay. Other groups have successfully performed pooling with similar numbers of samples but utilized wet storage of swabs (8, 14–16). Bogere et al. (22) also validated pooling of 10 samples in Uganda and discuss the necessity of sample pooling in their country where testing needs far exceed individual testing capacity. Technically it appears possible to use even larger pool sizes, especially if samples are hydrated individually and then aliquots of those individual suspensions are combined, as opposed to our approach (which uses fewer tubes) of hydrating them together. Indeed, known positives are reported to be detectable in pools of 50 (23) and 100 (7). However, as we have shown the cost of large pool sizes increases rapidly with percent positivity because of retesting. One potentially more cost-effective approach would be to have a three-tiered strategy (24), involving larger then smaller pools followed by individual testing, but that would require more labor and/or a customized automated workflow.

One clear drawback of pooled testing is that all individuals in positive pools need to be retested to identify the positive individual. A few approaches can be used to facilitate this: individuals from the positive pools can be asked to return for retesting or every individual provides two samples with the second swab stored for retesting. The former approach places the burden on individuals to return for retesting. Of course, having positive individuals return to testing sites increases the risk for transmission at the site and potentially during their commute—especially if the individual is relying on public or shared transport. Enhanced safety, distancing and cleaning measures, and potentially the use of separate dedicated retesting areas or sites, may mitigate some of this increased risk. A key element to retesting is that all individuals in a positive pool should be treated as potentially positive and should therefore employ appropriate quarantine measures. Importantly, retesting should be performed as soon as possible after the original test to further limit transmission but also to release negative individuals from quarantine in a timely fashion. We suggest that retesting be performed with a rapid antigen testing method, preferably in concert with a confirmatory PCR test, to obtain the infection status of individuals as soon as possible. It must be emphasized that the feasibility of the return visit approach diminishes with increasing disease prevalence due to increasing risk of transmission as well as increased logistical and financial burden of repeat testing. The latter approach of collecting two swabs (and storing one for retesting) places the burden on the screening organization to store and retrieve samples and requires substantial amounts of additional supplies to collect those samples. The benefits of this approach are that retesting can likely occur much faster and positive individuals are not required to return to the testing site—both factors that would likely reduce the risk for transmission. Despite the potential risks, we prefer the return visit approach because many institutions simply would not have the capacity to safely preserve and retrieve second swabs for retesting—but only when case rates remain sufficiently low so that the cost-benefits outweigh the potential risks associated with return visits.

We performed a cost-benefit analysis to better understand the potential savings that could be realized through pooled testing. Although fewer samples are processed, additional handling is required to prepare and process the pooled samples. Based on a sample pool size of eight and costs of $25 and $40 for individual and pooled tests, respectively, we estimate that savings from using a pooled method of testing vs. individual testing would exist for positivity rates under 10% ($19.88 in savings at 0.01% positivity).

It is worth noting that our observation of a low rate of improper test collection (absence of RNA on the swab, excessive material on the swab, swab inverted) among volunteers who collected their own samples (self-swab). In settings where a trained professional is collecting samples, we expect the rate to decrease dramatically. However, when individuals self-swab it will be impossible to detect individuals with repeat errors in collection methods e.g., those who are not swabbing appropriately because the other samples in the pool will mask it. Therefore, education of self-swabbing subjects will be critical.

A lack of in-person education deprives children of educational and well as social development (1, 25). Maintaining in-person school attendance during the COVID-19 pandemic will rely on early identification and containment of infectious individuals. We have validated a pooled Covid-19 testing method in a population consisting of University students, faculty, and staff with a minimum age of 18 years. The pooling method we present here is a simple, scalable way to reduce the cost of regular surveillance screening. Assuming that nasal sampling can be performed appropriately, especially amongst younger students, the method is likely also suitable for kindergarten−12th grade schools as well.

## Data Availability Statement

The original contributions presented in the study are included in the article/supplementary material, further inquiries can be directed to the corresponding author/s.

## Ethics Statement

The studies involving human participants were reviewed and approved by Institutional Review Board of Tufts University. The patients/participants provided their written informed consent to participate in this study.

## Author Contributions

CG and AM: study conception and design. AS, JC, and AR: human study. SG, NL, and BB: sample analyses. AS, JC, and CS: data analysis and interpretation. AS, JC, and CG: manuscript. All authors contributed to the article and approved the submitted version.

## Conflict of Interest

The authors declare that the research was conducted in the absence of any commercial or financial relationships that could be construed as a potential conflict of interest.

## Publisher's Note

All claims expressed in this article are solely those of the authors and do not necessarily represent those of their affiliated organizations, or those of the publisher, the editors and the reviewers. Any product that may be evaluated in this article, or claim that may be made by its manufacturer, is not guaranteed or endorsed by the publisher.
